# SLIMarray: Lightweight software for microarray facility management

**DOI:** 10.1186/1751-0473-1-5

**Published:** 2006-10-26

**Authors:** Bruz Marzolf, Pamela Troisch

**Affiliations:** 1 Institute for Systems Biology, 1441 N. 34^th ^Street, Seattle, Washington, USA

## Abstract

**Background:**

Microarray core facilities are commonplace in biological research organizations, and need systems for accurately tracking various logistical aspects of their operation. Although these different needs could be handled separately, an integrated management system provides benefits in organization, automation and reduction in errors.

**Results:**

We present *SLIMarray *(**S**ystem for **L**ab **I**nformation **M**anagement of Micro**array**s), an open source, modular database web application capable of managing microarray inventories, sample processing and usage charges. The software allows modular configuration and is well suited for further development, providing users the flexibility to adapt it to their needs. *SLIMarray Lite*, a version of the software that is especially easy to install and run, is also available.

**Conclusion:**

*SLIMarray *addresses the previously unmet need for free and open source software for managing the logistics of a microarray core facility.

## Background

Core facilities are a common paradigm in the design of academic, non-profit and commercial biological research organizations. In this model, multiple research groups utilize the specialized resources provided by core facilities, such as sequencing, genotyping and microarray services. Often there is a mechanism by which these facilities charge the individual research groups for the products and services they provide to them.

Managing a core facility typically involves keeping organized and up-to-date inventories of consumables, tracking samples processed, and recording charges to researchers for products and services provided to them. The considerable number of samples processed and the substantial charges that researchers incur necessitate a highly organized and accurate system for managing this information. A simple solution is to use Microsoft Excel for tracking these transactions, but this often requires redundant data entry into multiple spreadsheets, and is prone to error. Lab information management systems (LIMS) software addresses this problem by storing information in interrelated tables with more rigorous data entry mechanisms in place to prevent inaccuracies and reduce redundancy.

Numerous microarray databases exist [1-7], but these primarily address the needs of researchers by allowing them to store, manage and analyze their microarray data. Although some microarray databases provide LIMS capabilities, their features are designed for data management and do not address logistical needs such as inventory and usage fee tracking.

Here we present *SLIMarray *(**S**ystem for **L**ab **I**nformation **M**anagement of Micro**array**s), a database software application designed specifically for microarray core facilities. *SLIMarray *provides simple interfaces for managing inventory, sample processing and charging information, and automatically creates interrelated data records where appropriate.

## Implementation

### Architecture

**Figure 1:**
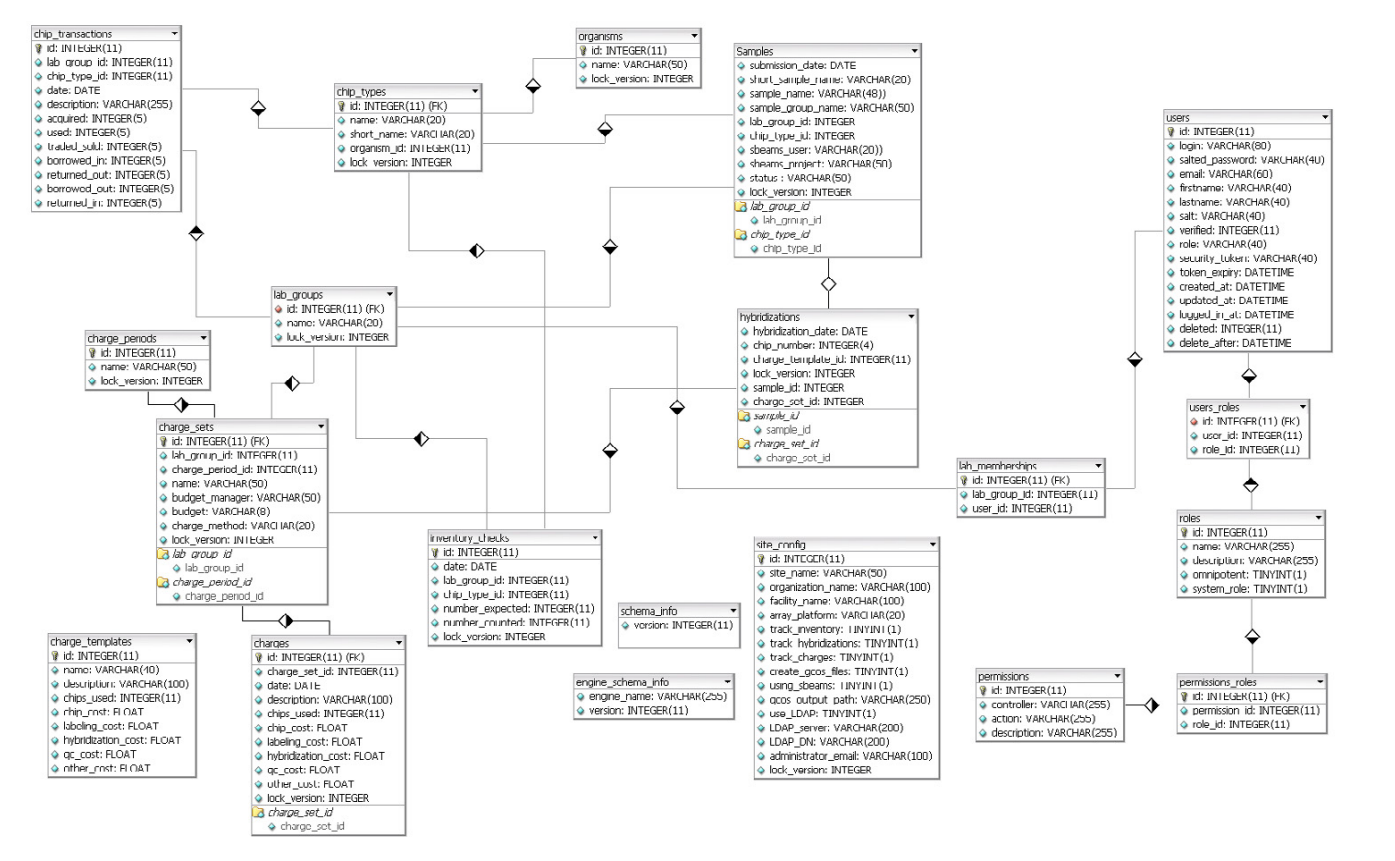
***SLIMarray *database schema**. A diagrammatic depiction of the tables used to store information in *SLIMarray *and the relationships among them.

*SLIMarray *is built using the Ruby on Rails [8] framework for database-backed web applications. Ruby on Rails uses a Model-View-Controller approach, where models are employed to programmatically interact with a database, controllers implement the application logic, and views specify the user interface. The database schema (Figure 1) includes tables containing information about microarray inventories, processing, charges, application configuration, lab groups, and user authentication and authorization. *SLIMarray *has been designed and tested to support both MySQL and SQLite, and should work, but has not been tested with PostgreSQL, IBM DB2, Oracle and Microsoft SQL Server. The Rails Engines, LoginEngine and UserEngine provide user authentication and authorization control. *SLIMarray *is deployed using a web server, and is accessed by any number and type of web browser clients.

### Customer interfaces

*SLIMarray *is primarily intended as a management tool for facility staff, but provides limited access to information relevant to facility customers. Accounts can be created for customers that allow them to submit and view the status of samples, and check their microarray inventories. These customer accounts restrict access to all other areas of *SLIMarray*.

### Microarray inventory management

Microarrays are costly, especially when acquired from commercial vendors, making inventory tracking important in ensuring that array investments are accounted for properly. *SLIMarray *allows tracking of multiple lab groups' inventories of different chip types, with records of every transaction such as purchasing or using microarrays. Microarrays are accounted for by using the inventory-checking interface, where users physically count their arrays and reconcile them with the inventory numbers in the database. Additionally, inventories are linked to hybridization management.

### Sample submission

Microarray core facilities collect information about samples, such as what type of array they should be hybridized to, which inventory the arrays should come from, and how the resulting data should be annotated. *SLIMarray *provides a sample submission interface for customers to directly enter their sample information into the database, ensuring that sample information is complete and as desired by the customer. The information required of the customer depends upon the software configuration, so that they are not required to provide unnecessary information.

### Hybridization entry

**Figure 2:**
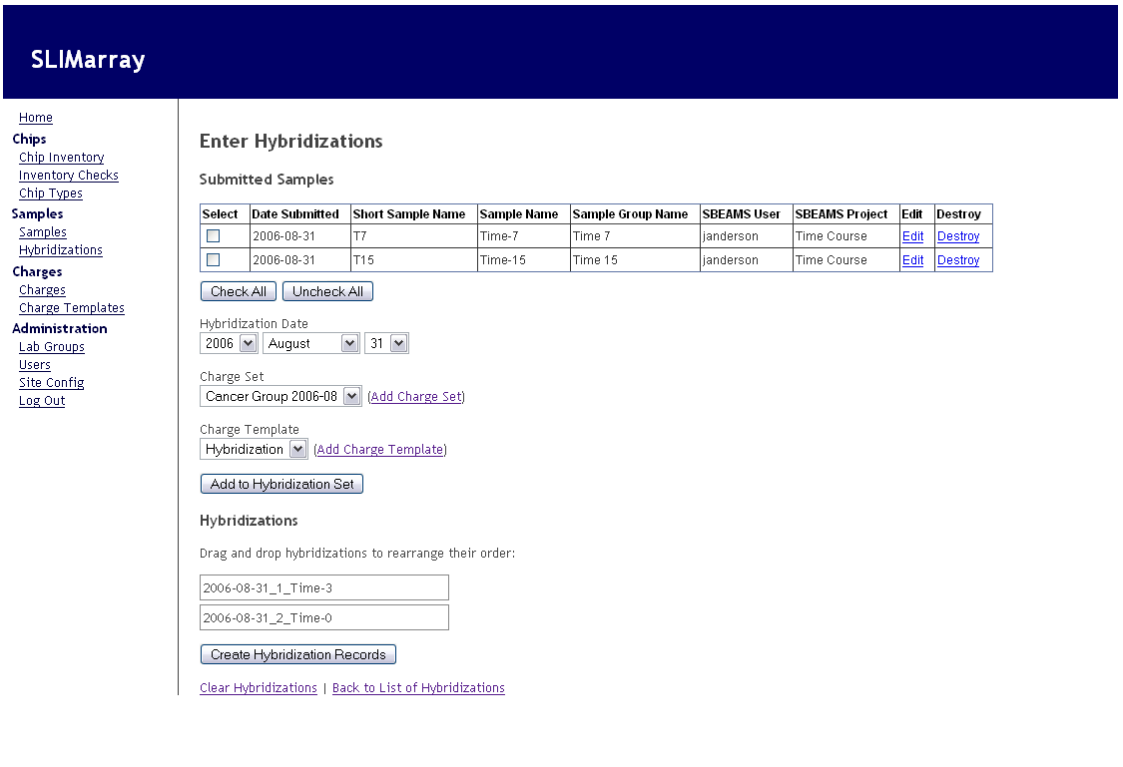
**Hybridization entry interface**. Screenshot showing a web browser view of the interface used to enter information about hybridizations. Other interfaces in *SLIMarray *are similar in appearance.

The hybridization entry interface (Figure 2) allows facility staff to create records of hybridizations performed, and automatically records accompanying changes to inventories and charges. The hybridization entry interface presents a list of submitted samples. The user selects all or a subset of these, and chooses options that determine how they will be charged. Once the user has compiled a list of samples to hybridize, they are able to reorder the list as desired. Upon creation of the hybridization records, the software produces inventory transactions that record the number of arrays used in the inventories of the appropriate lab groups. Charge records are created and filed under the appropriate lab group's set of charges for the most recent charge period. In addition, if Affymetrix samples are being recorded, files can be output for immediate and automated sample record generation in Affymetrix GeneChip Operating System (GCOS) software [9].

### Usage charge management

Facility usage charges can be tracked as a means of producing well-organized charge summaries to bill lab groups. Charges are separated into charge periods, for instance monthly periods if that is the billing cycle a facility uses. Within each charge period there can be any number of charge sets (collections of charges for a particular lab group and project). As described in the previous section, entry of hybridizations automatically records costs for the appropriate charge set. Interfaces for managing charge records are available separate from hybridization entry. Charge templates can be created to ease entry of common types of charges. Reports of charges can be output in both PDF and Microsoft Excel format.

### Security and administration

Users must log in to gain access to *SLIMarray*, and are authenticated either using encrypted passwords generated and stored in the database, or optionally by connecting to a Lightweight Directory Access Protocol (LDAP) server. Users may be assigned any number of roles determining their permissions for each interface in the application. Roles are provided for administrators, facility staff and facility customers.

Many of the application configuration settings are stored in the database and can be edited through the web interface, making external configuration files unnecessary. Administration of users and user permissions are also configurable through the application interfaces by users with administrative privileges.

### Modular configuration

*SLIMarray *is configurable in a modular fashion, allowing users to enable or disable functionality to suit the needs of their particular microarray facility. Each of the major types of information tracked, including inventory, hybridizations and charges, is optional. Disabling one or more of these modules will remove menu choices related to it, as well as portions of other modules' interfaces and functionality that are rendered irrelevant. Users choose whether their array platform is Affymetrix, non-Affymetrix or a combination of using both platforms. Other options include GCOS software sample generation, use of SBEAMS [4] as the downstream analysis database, and use of LDAP for user login authentication.

### Installation

The software is available in two formats, depending upon the needs of the facility installing it. The *SLIMarray Lite *distribution is installed simply by downloading an archive file from the *SLIMarray *website [10] and extracting its contents. This provides the entire application as a single executable file for either Linux or Windows, along with SQLite database files. The inherent limitations of *SLIMarray Lite *are that it can only use SQLite, it runs a pure-ruby web server, and it is not modifiable for further development.

The full *SLIMarray *software can either be downloaded as a distribution release, or obtained from the source code repository, both of which are available through the *SLIMarray *website [10]. The full *SLIMarray *installation can be configured for deployment with MySQL Server and Lighttpd HTTP Server, although other database and web server options are possible. Installation documentation for *SLIMarray *and *SLIMarray Lite *on both Windows and Linux platforms is detailed at the *SLIMarray *website [10].

A demonstration instance of *SLIMarray *has been made available through the *SLIMarray *website [10] to allow users to evaluate the software prior to downloading and installing it.

## Results and Discussion

### Initial preparation

After installation of *SLIMarray*, users set their configuration options through the web interface. Names for the web site, organization and facility may be specified, array platform options chosen, and different modules enabled or disabled. GCOS, SBEAMS and LDAP usage are enabled and configured through the web interface as well. These configuration options are described in the *SLIMarray Administration Guide *(Additional file 1).

Lab groups and chip types must then be created corresponding to the lab groups that will be served and the chip types that are used by those lab groups. Initial inventories are established by creating chip transactions to reflect the physical inventories at the time database tracking begins.

### Daily workflow

In conjunction with physically giving their samples to the microarray core facility, customers will use the sample submission interface to provide necessary information to the facility staff. Customer interfaces to *SLIMarray *are described in the *SLIMarray Customer Guide *(Additional file 3).

Prior to performing hybridizations, facility staff will create a hybridization list by selecting samples that their customers have submitted and adding some additional information. Upon completion of the hybridization records, *SLIMarray *creates chip transaction records accounting for the hybridized microarrays, and charges are produced detailing the costs incurred for those hybridizations. A printable table of hybridization information, which can be taped into a technician's lab notebook, is generated after the hybridization records are entered. Documentation of facility tasks is provided in the *SLIMarray Facility Staff Guide *(Additional file 2).

### Periodic tasks

Less frequent tasks include performing inventory checks and generating charge reports. Users may choose to establish a regular schedule for physically checking microarray inventories and reconciling them against the inventory records in the database.

At the end of each billing cycle users will edit and add charges as needed to the charge sets for each lab group and project, in addition to those created in tandem with hybridization data entry. After the charge sets for the current charge period are complete, users will use the PDF or Excel generation functions to produce documents that can be given to the lab groups and others involved with the billing process.

### Further development

As an open source application developed in the Ruby on Rails framework, *SLIMarray *is well suited for further enhancements by users seeking to alter or add software functionality.

## Conclusion

*SLIMarray *is a free and open source tool for microarray core facility management. It is easy to install without advanced knowledge of computers. It is highly configurable, modular and extensible, allowing users to benefit from the software modules they need, and modify it to suit their needs.

## Availability and requirements

• **Project name: **SLIMarray

• **Project home page: **

• **Operating system: **Windows, Linux

• **Programming language: **Ruby on Rails

• **Other requirements:**

○ MySQL Server (optional)

○ Lighttpd or Apache HTTP Server (optional)

• **License: **GNU General Public License version 2

• **Any restrictions to use by non-academics: **None

## Authors' contributions

BM conceived the project, designed and implemented the software, documented the installation procedure, wrote the administrator documentation and drafted the manuscript. PT contributed to and edited the manuscript, tested the software and installation procedures, and wrote the facility staff and customer documentation.

## Supplementary Material

Additional file 1 ***SLIMarray Administration Guide***. The *SLIMarray Administration Guide *describes software configuration and managing user permissions (functions that are accessible to users with Administrator privileges). Click here for file

Additional file 3 ***SLIMarray Customer Guide***. Sample submission and management, as well as microarray inventory browsing, are described in the *SLIMarray Customer Guide*. Click here for file

Additional file 2 ***SLIMarray Facility Staff Guide***. Daily workflow and periodic tasks performed by facility staff are covered in the *SLIMarray Facility Staff Guide*. Click here for file
