# Supplementary material for: SLIMarray: Lightweight software for microarray facility management
Source: Source Code Biol Med. 2006 Oct 26;1:5. doi: 10.1186/1751-0473-1-5 (PMC1636632; doi:10.1186/1751-0473-1-5)
Supplement: Additional file 1 — SLIMarray Administration Guide. The SLIMarray Administration Guide describes software configuration and managing user permissions (functions that are accessible to users with Administrator privileges). [file 1751-0473-1-5-S1.pdf]

INSTITUTE FOR Systems Biology

Revolutionizing science. Enhancing life.

SLIMarray Administrator Guide

Bruz Marzolf

September 7, 2006

Table of Contents

1. Introduction 3
2. Initial Login and Securing Administrator Account 3
  2.1. First Login 3
  2.2. Changing Administrator Password 3
3. User and Role Management 3
  3.1. Creating New Users 3
  3.2. Editing User Accounts 3
4. Web Configuration 5
  4.1. Configuration Interface 5
  4.2. Configuration Details 5
5. GCOS Software Configuration 6
  5.1. GCOS Automatic Sample Loading 6
  5.2. GCOS Template Configuration 6

1. Introduction

This guide describes administration of SLIMarray and SLIMarray Lite. It assumes that SLIMarray has already been successfully installed. If this is not the case, the installation documentation can be found at http://slimarray.systemsbiology.net/doc/files/README.html.

2. Initial Login and Securing Administrator Account

2.1. First Login

Open a web browser and navigate to the web address of your SLIMarray installation. If you're doing this on the computer where you installed the software, and you're using WEBrick, it will be http://localhost:3000.

Enter the default administrator Login ID of 'admin' and Password 'admin', and click **Login**. This will take you to the SLIMarray Administrator Home page. The menu on the left provides access to all of the functionality available in SLIMarray.

2.2. Changing Administrator Password

It is advisable for security purposes to change the default administrator password. To accomplish this, first select **Users** from the left-hand menu.

A listing of users will be displayed, in this case the only one being the default 'admin' user. Select the **Edit** link on the right-hand side of the 'admin' user listing.

In the Password section, enter a new password of your choice in both the Password and Password Confirmation fields. Click the **Change password** button.

3. User and Role Management

3.1. Creating New Users

As in section **2.2**, start by selecting **User** from the left-hand menu. Below the listing of users, choose the **New User** link.

This presents a form, requiring information about the user's name, login, email and password. The email is important since the forgotten password function will send an email to this address, allowing users to reset their own passwords in case they lose them. Once all of the information has been entered, choose **Create User**.

3.2. Editing User Accounts

By default, newly created users have access to very little of SLIMarray, so users must be modified by an administrator to assign privileges. To edit a user, select **User** on the left-hand menu, and then choose the **Edit** link next to the user you would like to edit.

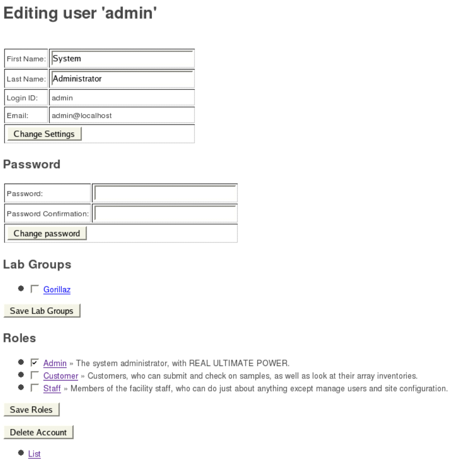

Figure 1 - Editing a User

The first section of user editing allows changes to the user's name, but neither login nor email can be changed. After making desired name changes, choose **Change Settings** to update the name information.

The Password section allows a new password to be assigned to a user. Enter the new password for the user in both the Password and Password Confirmation fields, and select **Change password**.

Using the Lab Groups section, a user can be assigned to any lab groups for which they ought to be able to see array inventories and submit samples. Select the checkbox next to any lab groups the user belongs to, and then **Save Lab Groups**.

User may have any number of Roles. Roles define what interfaces and actions a user has access to, and generally should not need to be altered. Administrators needing to have complete access should have the Admin role, facility staff should have the Staff role, and customers of the microarray facility should have the Customer role. Table 1 describes the differences between the three types of roles. Select the checkbox to whichever roles the user should have, and choose **Save Roles**.

Access Area	Customer	Staff	Admin
Chip Inventories	✓ (Within Lab Group)	✓	✓
Samples	✓ (Within Lab Group)	✓	✓
Inventory Checks		✓	✓
Chip Types		✓	✓
Hybridizations		✓	✓
Charges		✓	✓
Charge Templates		✓	✓
Lab Groups		✓	✓
Users			✓
Site Configuration			✓

Table 1 – Capabilities of different Roles

4. Web Configuration

4.1. Configuration Interface

After installation, essentially all of the SLIMarray application configuration settings can be accessed through the software's web interface.

Configuration settings are reached by selecting **Site Config** on the left-hand menu.

4.2. Configuration Details

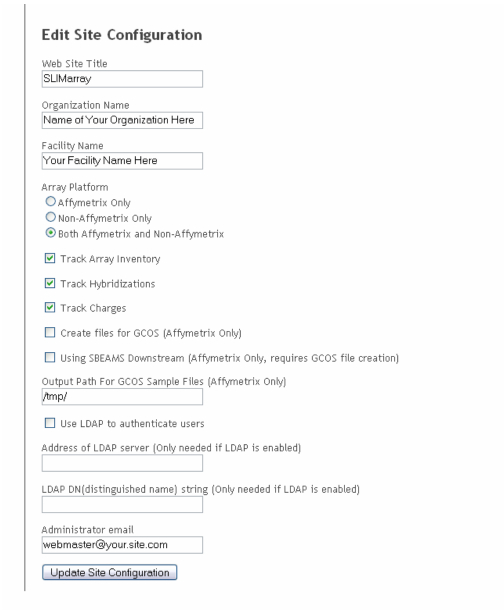

Figure 2 - Configuration Interface

The **Site Config** interface presents a list options (Figure 2), described here one-by-one:

- **Web Site Title:** The name you would like to appear in the title bar of the application and as the site title in users' web browsers.
- **Organization Name:** Name for the organization where the software is deployed. This name appears in the PDF reports that are produced in the Charges area.
- **Facility Name:** Name of the microarray facility, to appear on the PDF charge reports along with the **Organization Name**.
- **Array Platform:** Indicates whether usage will be exclusively Affymetrix, exclusively non-Affymetrix or a combination of the two. This affects what information is gathered in various forms.
- **Track Array Inventory:** Determines whether the **Chip Inventory** and **Inventory Checks** menu choices and interfaces will be available, and whether inventory transactions will be recorded when hybridization records are created.
- **Track Hybridizations**: Determines whether the **Samples** and **Hybridizations** menu choices and interfaces will be available.
- **Track Charges:** Determines whether the **Charges** and **Charge Templates** interfaces will be available, and whether charge records are recorded when hybridizations are created.

- **Create files for GCOS:** If this is selected, additional information will be obtained by the software during the sample submission step, and files will be output (see **Output Path for GCOS…** option) for automatic sample creation by GCOS.
- **Using SBEAMS Downstream:** If this option is selected, additional sample information is collected, enabling it to be propagated through GCOS and loaded into SBEAMS (see http://www.sbeams.org/Microarray). This requires a small configuration change in the GCOS software (see section **5.2**).
- **Output Path for GCOS Sample Files:** Used in conjunction with the **Create files for GCOS** option, this path indicates where the sample information file will be output. If this directory is set up to be scanned by the Affymetrix Importer tool, sample/experiment information will be automatically loaded into GCOS (see sections **5.1** and **5.2** for instructions on how to configure GCOS).
- **Use LDAP to authenticate users:** If this option is enabled, SLIMarray will attempt to use the information in the subsequent two fields to authenticate users via an LDAP server. This requires use of the OpenLDAP software, and has only been tested in a Linux environment.
- **Address of LDAP Server:** The web address of your LDAP server, often something like ldap.yourdomain.com.
- **LDAP DN (distinguished name) string:** The string that is used when connecting to the LDAP server, which may be something like "cn=users,dc=yourdomain,dc=com". The software will append "cn=<user full name>" to this string. Unfortunately, LDAP configurations are not standardized well enough that this will work for all LDAP servers.
- **Administrator email:** This is used for emails from the software to users for forgotten password resets and to inform users of major changes to their accounts, such as deletion of a user.

5. GCOS Software Configuration

The GCOS software must be configured properly to automatically load sample information, and to handle propagation of SBEAMS information.

5.1. GCOS Automatic Sample Loading

GCOS can be configured to scan a specified directory for new sample information files by installing the GCOS *Sample/Experiment Object Importer*, which can be downloaded from http://www.affymetrix.com/support/developer/tools/devnettools.affx. Install the software on your Affymetrix instrument computer, and configure it to point to a directory that you have configured in SLIMarray as the **Output Path for GCOS Sample Files** (see section **4.2**).

5.2. GCOS Template Configuration

In order to properly load the sample information file through the *Sample/Experiment Object Importer*, it is necessary to create a template to hold all the desired information.

On your Affymetrix instrument computer, open *GCOS Manager*, and go to the **Templates** tab. In the tree on the left-hand side of the screen, right-click on **Sample Templates** and choose **Create**. Enter 'AffyCore' as the template name. Add a single attribute to the template, with Name of 'Array User Name', Type of 'String' and Required set to 'Yes'. Choose **Apply**.
