# Supplementary material for: SLIMarray: Lightweight software for microarray facility management
Source: Source Code Biol Med. 2006 Oct 26;1:5. doi: 10.1186/1751-0473-1-5 (PMC1636632; doi:10.1186/1751-0473-1-5)
Supplement: Additional file 3 — SLIMarray Customer Guide. Sample submission and management, as well as microarray inventory browsing, are described in the SLIMarray Customer Guide. [file 1751-0473-1-5-S3.pdf]

INSTITUTE FOR Systems Biology

Revolutionizing science. Enhancing life.

SLIMarray

Customer Guide

by Pamela Troisch

September 7, 2006

Table of Contents

1 Access SLIMarray as a Customer User 3
1.1 Description 3
1.2 Login 3
2 Chips 3
2.1 Chip Inventory 3
3 Samples 4
3.1 Status of Submitted Samples 4
3.2 Submitting New Samples 4
4 Exit SLIMarray 5
4.1 Log out 5

1 Access SLIMarray as a Customer User

1.1 Description

SLIMarray users with Customer permissions have access to view their Lab Group's chip inventory and the ability to enter samples for hybridization.

1.2 Login

Login with your username and password as set up by your SLIMarray administrator. This will open the home page (see Figure 1). The home page will display a table of samples that have been entered for future hybridization under **Samples Submitted for Hybridization**. Under the title **Array Inventories** the type of chips available to your group are listed alongside the number of chips for each type on hand.

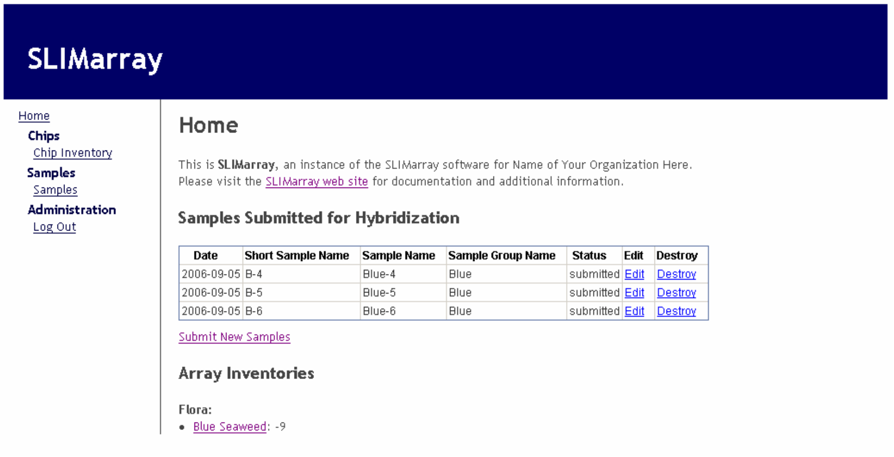

SLIMarray

Home
Chips
Chip Inventory
Samples
Samples
Administration
Log Out

Home

This is **SLIMarray**, an instance of the SLIMarray software for Name of Your Organization Here.
Please visit the SLIMarray web site for documentation and additional information.

Samples Submitted for Hybridization

Date	Short Sample Name	Sample Name	Sample Group Name	Status	Edit	Destroy
2006-09-05	B-4	Blue-4	Blue	submitted	Edit	Destroy
2006-09-05	B-5	Blue-5	Blue	submitted	Edit	Destroy
2006-09-05	B-6	Blue-6	Blue	submitted	Edit	Destroy

Submit New Samples

Array Inventories

Flora:
- Blue Seaweed: -9

Figure 1 - Successful Login

2 Chips

2.1 Chip Inventory

Click on **Chip Inventory** under the **Chips** heading; your lab group will be listed with all available chip types. Click on a chip type to view a detailed list of current transactions and a table of running totals (see Figure 2).

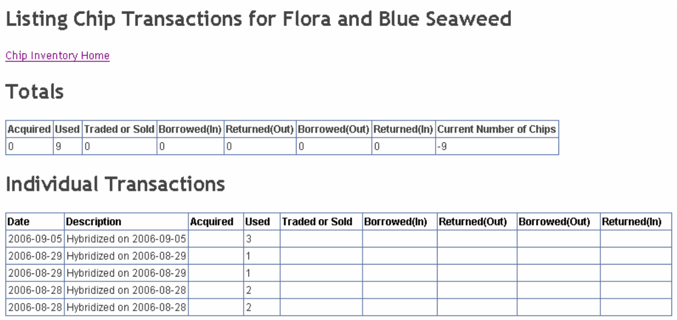

Listing Chip Transactions for Flora and Blue Seaweed

Chip Inventory Home

Totals

Acquired	Used	Traded or Sold	Borrowed(In)	Returned(Out)	Borrowed(Out)	Returned(In)	Current Number of Chips
0	9	0	0	0	0	0	-9

Individual Transactions

Date	Description	Acquired	Used	Traded or Sold	Borrowed(In)	Returned(Out)	Borrowed(Out)	Returned(In)
2006-09-05	Hybridized on 2006-09-05		3					
2006-08-29	Hybridized on 2006-08-29		1					
2006-08-29	Hybridized on 2006-08-29		1					
2006-08-28	Hybridized on 2006-08-28		2					
2006-08-28	Hybridized on 2006-08-28		2					

Figure 2 - Chip Inventory

3 Samples

3.1 Status of Submitted Samples

Under the heading **Samples**, click **Samples**. A list of all samples entered by your lab group will be displayed in a table showing the status of each individual sample (see Figure 2). You have the ability to **Edit** or **Destroy** the record of a particular sample.

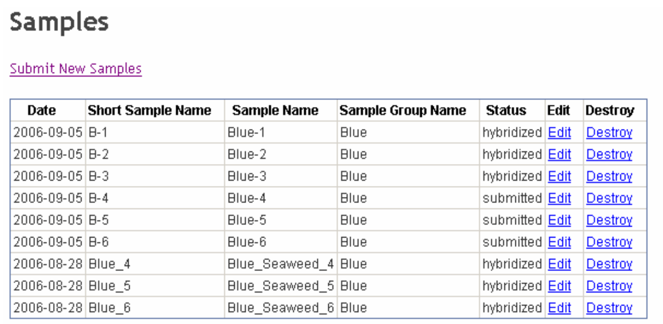

Samples

Submit New Samples

Date	Short Sample Name	Sample Name	Sample Group Name	Status	Edit	Destroy
2006-09-05	B-1	Blue-1	Blue	hybridized	Edit	Destroy
2006-09-05	B-2	Blue-2	Blue	hybridized	Edit	Destroy
2006-09-05	B-3	Blue-3	Blue	hybridized	Edit	Destroy
2006-09-05	B-4	Blue-4	Blue	submitted	Edit	Destroy
2006-09-05	B-5	Blue-5	Blue	submitted	Edit	Destroy
2006-09-05	B-6	Blue-6	Blue	submitted	Edit	Destroy
2006-08-28	Blue_4	Blue_Seaweed_4	Blue	hybridized	Edit	Destroy
2006-08-28	Blue_5	Blue_Seaweed_5	Blue	hybridized	Edit	Destroy
2006-08-28	Blue_6	Blue_Seaweed_6	Blue	hybridized	Edit	Destroy

Figure 2 - Submitted Samples

3.2 Submitting New Samples

To submit new samples click on **Submit New Samples** at the top of the table (this can also be done by clicking **Submit New Samples** on the home page). Fill in the fields provided with the sample information (see Figure 3).

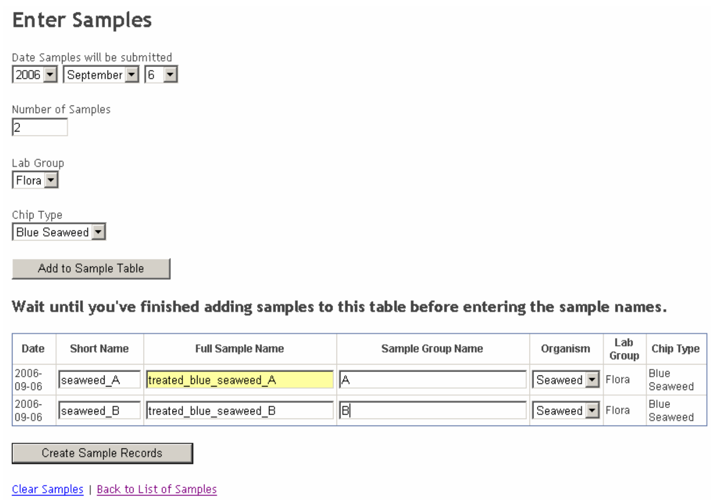

Figure 3 – Submitting Samples

The fields of information requested are dependant on the site configurations determined by the administrator. Click on the **Add to Sample Table** button when completed. Repeat with remaining batches of samples.

Do not start filling out the **Sample Table** until all the samples to be submitted have been added to the table.

After the **Add to Sample Table** button is clicked information on each sample will be requested. Sample names cannot have symbols in them, such as + or /, or spaces, instead use a dash or an underscore. If samples are entered incorrectly an error message will pop up prompting you to make appropriate corrections.

Once entered, click **Create Sample Records.** The software will let you know that the records have been successfully created (see Figure 4).

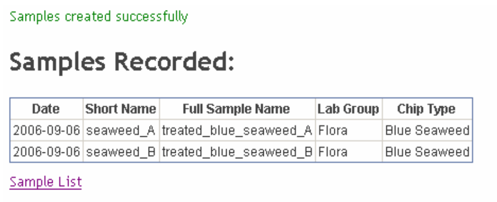

Samples created successfully

Samples Recorded:

Date	Short Name	Full Sample Name	Lab Group	Chip Type
2006-09-06	seaweed_A	treated_blue_seaweed_A	Flora	Blue Seaweed
2006-09-06	seaweed_B	treated_blue_seaweed_B	Flora	Blue Seaweed

Sample List

Figure 4 – Entered Samples

The samples will now be listed in the **Sample List** with status 'submitted'. They will also appear on the home page in the table under the heading **Samples Submitted for Hybridization**, until they have been selected for hybridization by the facility.

4 Exit SLIMarray

4.1 Log out

Click **Log Out** to exit SLIMarray and return to the login page.
