# Supplementary material for: SLIMarray: Lightweight software for microarray facility management
Source: Source Code Biol Med. 2006 Oct 26;1:5. doi: 10.1186/1751-0473-1-5 (PMC1636632; doi:10.1186/1751-0473-1-5)
Supplement: Additional file 2 — SLIMarray Facility Staff Guide. Daily workflow and periodic tasks performed by facility staff are covered in the SLIMarray Facility Staff Guide. [file 1751-0473-1-5-S2.pdf]

INSTITUTE FOR
Systems
Biology
Revolutionizing science. Enhancing life.

SLIMarray

Facility Staff Guide

by Pamela Troisch
September 7, 2006

Table of Contents

1 Access SLIMarray as a Facility Staff User 3
1.1 Description 3
1.2 Login 3
2 Track Arrays 3
2.1 Chip Inventory 3
2.2 Inventory Checks 5
2.3 Creating and Editing Chip Types 6
3 Samples 7
3.1 Entering Samples to be Hybridized 7
4 Hybridizations 8
4.1 Selecting Samples to be Hybridized 8
5 Charges 10
5.1 Charge Templates 10
5.2 Charge Periods 11
5.3 Charge Sets 11
6 Lab Groups 13
6.1 Creating and Changing lab groups 13
7 Exit SLIMarray 13
7.1 Log out 13

1 Access SLIMarray as a Facility Staff User

1.1 Description

SLIMarray users with Facility Staff permissions have access to daily workflow tasks such as checking chip inventories, entering hybridizations, and monitoring charges. A Facility Staff user is, however, unable to perform administration functions such as manipulating users and changing the SLIMarray configuration.

1.2 Login

Login with your username and password as set up by your SLIMarray administrator. This will open the home page (see Figure 1).

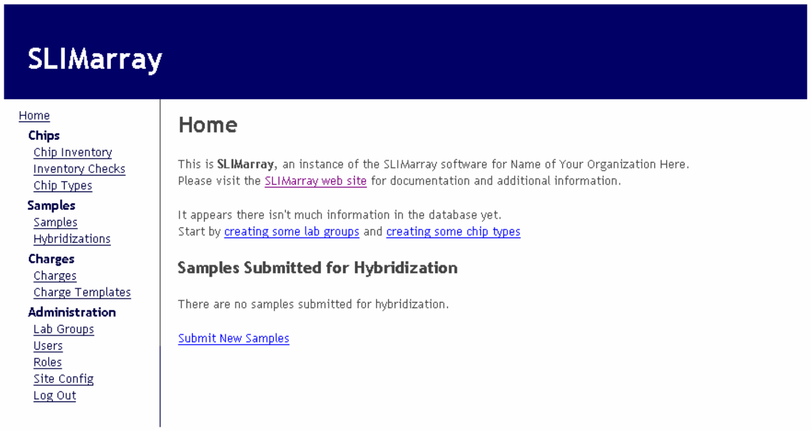

Figure 1 - Successful Login

If this is the first time logging in, the home page will prompt you to begin by entering lab groups and chip types.

Click on **creating some lab groups**; this will allow you to enter the name of a lab group. When finished click on the **Create** button and repeat for the remaining lab groups. Click on **creating some chip types** and fill in the requested fields with the name of the chip type, a short name (if it is an Affy array use the abbreviated name provided by Affymetrix), and enter the organism.
When finished hit the **Create** button.

2 Track Arrays

2.1 Chip Inventory

Click on **Chip Inventory** under the **Chips** heading; a list of lab groups will appear in a column with the chip types listed for each lab group (see Figure 2).

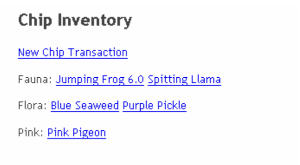

Figure 2 - Chip Inventory

Locate an individual lab group and select the chip type to track (see Figure 3).

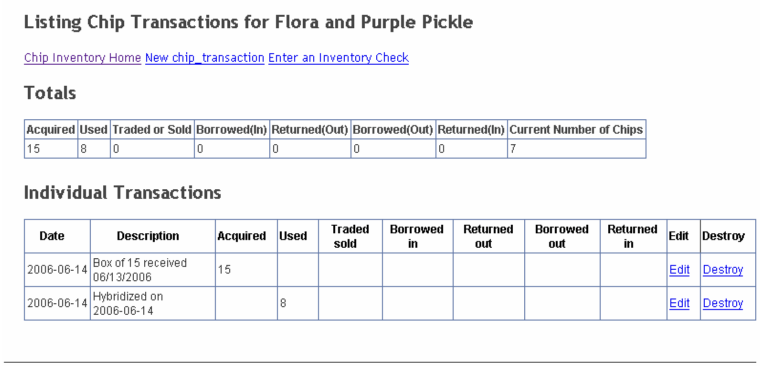

Listing Chip Transactions for Flora and Purple Pickle

Chip Inventory Home New chip_transaction Enter an Inventory Check

Totals

Acquired	Used	Traded or Sold	Borrowed(In)	Returned(Out)	Borrowed(Out)	Returned(In)	Current Number of Chips
15	8	0	0	0	0	0	7

Individual Transactions

Date	Description	Acquired	Used	Traded sold	Borrowed in	Returned out	Borrowed out	Returned in	Edit	Destroy
2006-06-14	Box of 15 received 06/13/2006	15							Edit	Destroy
2006-06-14	Hybridized on 2006-06-14		8						Edit	Destroy

Figure - 3 Tracking a Chip Inventory

A summary table of **Totals** lists the chips that have been acquired, used, traded, borrowed and returned, and the current number of chips that are available for hybridizations. A comprehensive table itemizing **Individual Transactions** is displayed under the summary table; it shows the date when a transaction occurred, the description of the transaction, the number of chips that have been acquired, used, or otherwise deployed.

To enter a transaction click on **New Chip Transaction** and fill in the appropriate fields to record the type of transaction (see Figure 4).

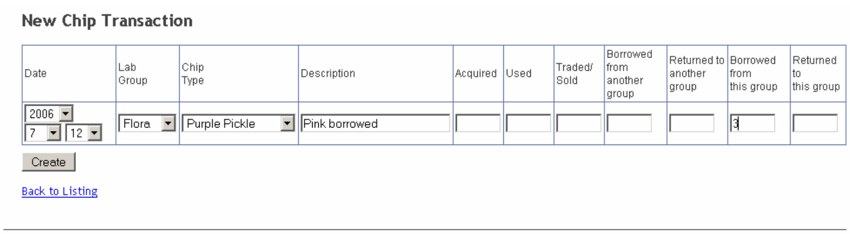

Figure 4 - Entering a New Chip Transaction

Hit the **Create** button when complete; the new transaction will be accounted for in both tables (see Figure 5).

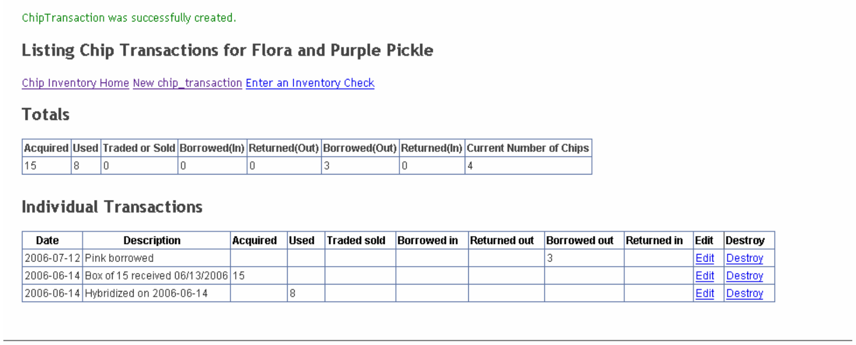
ChipTransaction was successfully created.

Listing Chip Transactions for Flora and Purple Pickle

Chip Inventory Home New chip_transaction Enter an Inventory Check

Totals

Acquired	Used	Traded or Sold	Borrowed(In)	Returned(Out)	Borrowed(Out)	Returned(In)	Current Number of Chips
15	8	0	0	0	3	0	4

Individual Transactions

Date	Description	Acquired	Used	Traded sold	Borrowed in	Returned out	Borrowed out	Returned in	Edit	Destroy
2006-07-12	Pink borrowed						3		Edit	Destroy
2006-06-14	Box of 15 received 06/13/2006	15							Edit	Destroy
2006-06-14	Hybridized on 2006-06-14		8						Edit	Destroy

Figure 5 - A Successful Chip Transaction

2.2 Inventory Checks

Inventory checks consist of physically counting the number of all the different types of arrays available for each group.

To enter a new inventory check click **Inventory Checks** and then **New Inventory Check**, or in **Chip Inventory** click **Enter an Inventory Check** (see Figure 6).

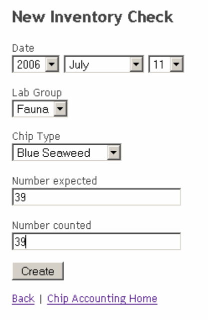

Figure - 6 Entering a New Inventory Check

Select the lab group and the chip type, and adjust the date to the day the chips were counted. In the **Number Expected** field enter the number of chips listed in **Chip Inventory** under **Current Number of Chips** in the **Totals** table and compare with the number entered into the field **Number Counted**. Click the **Create** button and a list of inventory checks will be displayed including the most recently entered one (see Figure 7).

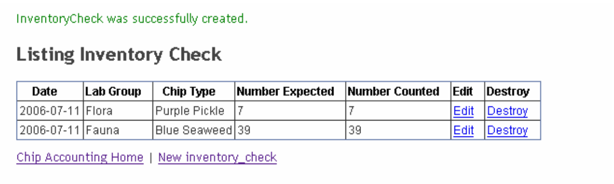
InventoryCheck was successfully created.

Listing Inventory Check

Date	Lab Group	Chip Type	Number Expected	Number Counted	Edit	Destroy
2006-07-11	Flora	Purple Pickle	7	7	Edit	Destroy
2006-07-11	Fauna	Blue Seaweed	39	39	Edit	Destroy

Chip Accounting Home | New inventory_check

Figure 7 - A Successful Inventory Check

The chip numbers should match, if not examine the individual transactions and investigate the discrepancy. The inventory check can be edited once the discrepancy is resolved by using the **Edit** link. To delete a record click the **Destroy** link.

2.3 Creating and Editing Chip Types

Click on **Chip Types** and a list of previously entered chips will be displayed (see Figure 8).

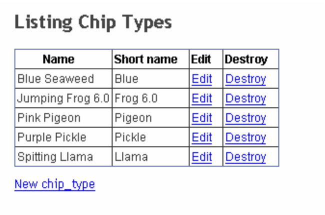
Listing Chip Types

Name	Short name	Edit	Destroy
Blue Seaweed	Blue	Edit	Destroy
Jumping Frog 6.0	Frog 6.0	Edit	Destroy
Pink Pigeon	Pigeon	Edit	Destroy
Purple Pickle	Pickle	Edit	Destroy
Spitting Llama	Llama	Edit	Destroy

New chip_type

Figure 8 - A List of Chip Types

To add a new type of chip click on **New chip _type** at the bottom of the list (see Figure 9).

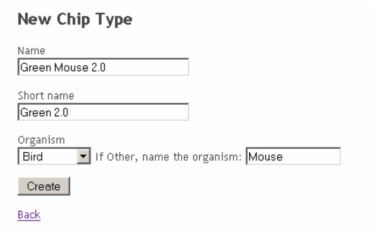

Figure 9 - Entering a New Chip Type

Enter the name of the chip and a short name to identify the chip, and then choose the organism from the drop down menu (if it is not present enter it in the field provided). Note: If using the Affymetrix system the short name should refer to the short name provided by Affymetrix.

When done click the **Create** button; the list of chip types will re-appear with the new chip included (see Figure 10).

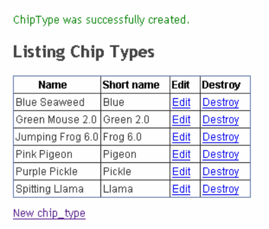
ChipType was successfully created.

Listing Chip Types

Name	Short name	Edit	Destroy
Blue Seaweed	Blue	Edit	Destroy
Green Mouse 2.0	Green 2.0	Edit	Destroy
Jumping Frog 6.0	Frog 6.0	Edit	Destroy
Pink Pigeon	Pigeon	Edit	Destroy
Purple Pickle	Pickle	Edit	Destroy
Spitting Llama	Llama	Edit	Destroy

New chip_type

Figure 10 - A Successful Chip Type Addition

To edit a chip type click on **Edit**; this will allow you to change the **Name**, **Short name** and the **Organism**, similar to entering a new chip type.

To remove a chip type click **Destroy**. A new window will pop up listing the hybridizations, inventory checks, and chip transactions related to the chip type and prompting you to confirm or abandon your decision (see Figure 11).

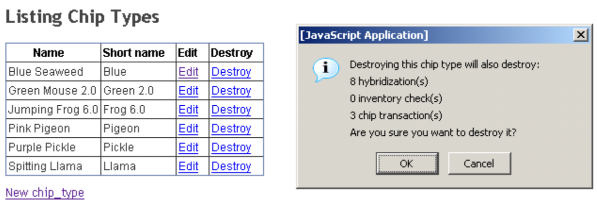

Figure 11 - Warning Before Selecting to Destroy a Chip Type

3 Samples

3.1 Entering Samples to be Hybridized

Under the heading **Samples**, click **Samples**. A list of samples submitted for hybridization will be displayed. Click on **Submit New Samples** at the top of the table. Fill in the fields provided with the sample information (see Figure 12).

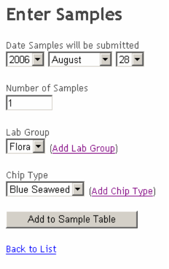

Figure 12 - Submitting Samples for Hybridization

The fields of information requested are dependant on the site configurations determined by the administrator. Click on the **Add to Sample Table** button when completed. Repeat with remaining batches of samples.

Do not start filling out the **Sample Table** until all the samples to be submitted have been added to the table.

After the **Add to Sample Table** button is clicked information on each sample will be requested (see Figure 13). Sample names cannot have characters in them, such as + or /, or spaces, instead use a dash or an underscore. If samples are entered incorrectly an error message will pop up prompting you to make appropriate corrections.

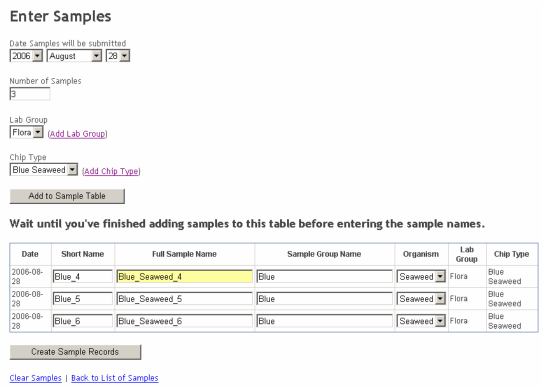

Enter Samples

Date Samples will be submitted
2006 | August | 28

Number of Samples
3

Lab Group
Flora (Add Lab Group)

Chip Type
Blue Seaweed (Add Chip Type)

Add to Sample Table

Wait until you've finished adding samples to this table before entering the sample names.

Date	Short Name	Full Sample Name	Sample Group Name	Organism	Lab Group	Chip Type
2006-08-28	Blue_4	Blue_Seaweed_4	Blue	Seaweed	Flora	Blue Seaweed
2006-08-28	Blue_5	Blue_Seaweed_5	Blue	Seaweed	Flora	Blue Seaweed
2006-08-28	Blue_6	Blue_Seaweed_6	Blue	Seaweed	Flora	Blue Seaweed

Create Sample Records

Clear Samples | Back to List of Samples

Figure 13 - Sample Information to be Entered

Once entered, the samples will appear on the home page in a table under the heading **Samples Submitted for Hybridization** (see Figure 14).

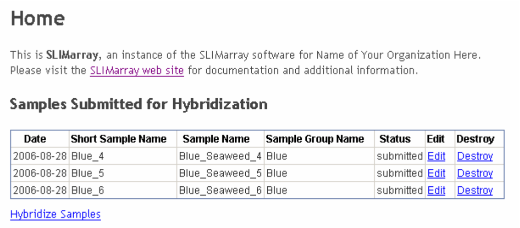

Home

This is SLIMarray, an instance of the SLIMarray software for Name of Your Organization Here.
Please visit the SLIMarray web site for documentation and additional information.

Samples Submitted for Hybridization

Date	Short Sample Name	Sample Name	Sample Group Name	Status	Edit	Destroy
2006-08-28	Blue_4	Blue_Seaweed_4	Blue	submitted	Edit	Destroy
2006-08-28	Blue_5	Blue_Seaweed_5	Blue	submitted	Edit	Destroy
2006-08-28	Blue_6	Blue_Seaweed_6	Blue	submitted	Edit	Destroy

Hybridize Samples

Figure - 14 Samples Submitted for Hybridization Displayed on the Home Page

4 Hybridizations

4.1 Selecting Samples to be Hybridized

Under the header **Samples** click on **Hybridizations**, a list of all previous hybs will be displayed, with the most recent ones at the beginning (see Figure 15).

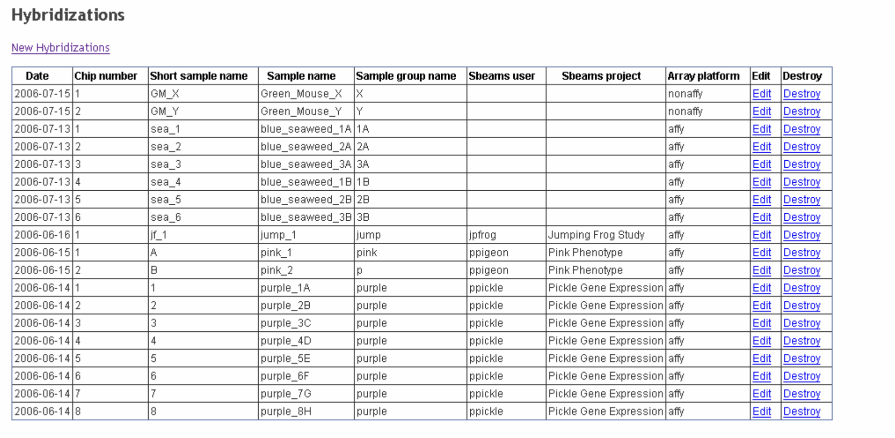

Hybridizations

New Hybridizations

Date	Chip number	Short sample name	Sample name	Sample group name	Sbeams user	Sbeams project	Array platform	Edit	Destroy
2006-07-15	1	GM_X	Green_Mouse_X	X			nonaffy	Edit	Destroy
2006-07-15	2	GM_Y	Green_Mouse_Y	Y			nonaffy	Edit	Destroy
2006-07-13	1	sea_1	blue_seaweed_1A	1A			affy	Edit	Destroy
2006-07-13	2	sea_2	blue_seaweed_2A	2A			affy	Edit	Destroy
2006-07-13	3	sea_3	blue_seaweed_3A	3A			affy	Edit	Destroy
2006-07-13	4	sea_4	blue_seaweed_1B	1B			affy	Edit	Destroy
2006-07-13	5	sea_5	blue_seaweed_2B	2B			affy	Edit	Destroy
2006-07-13	6	sea_6	blue_seaweed_3B	3B			affy	Edit	Destroy
2006-06-16	1	jf_1	jump_1	jump	jpfrog	Jumping Frog Study	affy	Edit	Destroy
2006-06-15	1	A	pink_1	pink	ppigeon	Pink Phenotype	affy	Edit	Destroy
2006-06-15	2	B	pink_2	p	ppigeon	Pink Phenotype	affy	Edit	Destroy
2006-06-14	1	1	purple_1A	purple	ppickle	Pickle Gene Expression	affy	Edit	Destroy
2006-06-14	2	2	purple_2B	purple	ppickle	Pickle Gene Expression	affy	Edit	Destroy
2006-06-14	3	3	purple_3C	purple	ppickle	Pickle Gene Expression	affy	Edit	Destroy
2006-06-14	4	4	purple_4D	purple	ppickle	Pickle Gene Expression	affy	Edit	Destroy
2006-06-14	5	5	purple_5E	purple	ppickle	Pickle Gene Expression	affy	Edit	Destroy
2006-06-14	6	6	purple_6F	purple	ppickle	Pickle Gene Expression	affy	Edit	Destroy
2006-06-14	7	7	purple_7G	purple	ppickle	Pickle Gene Expression	affy	Edit	Destroy
2006-06-14	8	8	purple_8H	purple	ppickle	Pickle Gene Expression	affy	Edit	Destroy

Figure - 15 An Up-to-date List of Hybridizations Performed

Click on **New Hybridizations**, or on the home page click **Hybridizations** at the bottom of the table of samples submitted for hybridizations. Select the individual samples to be hybridized (see Figure 16).

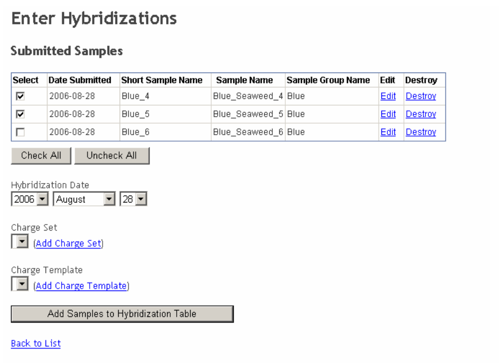

Figure - 16 Select Samples to be Hybridized

Enter the **Hybridization Date**, select a **Charge Set** and a **Charge Template**. Click on the **Add Samples to the Hybridization Table** button. The samples will now be listed under **Hybridizations**.

By clicking and dragging a sample within the list will rearrange the order, changing the chip number listed after the date associated with the sample depending on the position in the list (see Figure 17).

Hit the **Create Hybridization Records** button when complete.

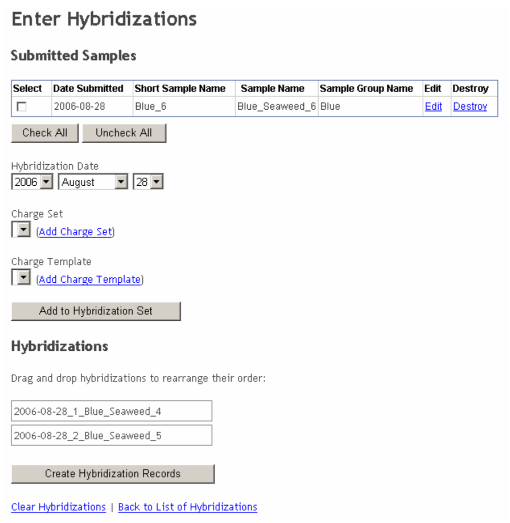

Figure - 17 A list of Planned Hybridizations

A printable table of current hybridizations will be displayed (see Figure 18).

Hybridizations were successfully created.

Hybridizations Recorded:

Date	Chip Number	Short Name	Full Sample Name	Lab Group	Chip Type
2006-07-13	1	sea_1	blue_seaweed_1A	Fauna	Blue Seaweed
2006-07-13	2	sea_2	blue_seaweed_2A	Fauna	Blue Seaweed
2006-07-13	3	sea_3	blue_seaweed_3A	Fauna	Blue Seaweed
2006-07-13	4	sea_4	blue_seaweed_1B	Fauna	Blue Seaweed
2006-07-13	5	sea_5	blue_seaweed_2B	Fauna	Blue Seaweed
2006-07-13	6	sea_6	blue_seaweed_3B	Fauna	Blue Seaweed

Hybridization List

Figure - 18 Printable Hybridization Record

5 Charges

5.1 Charge Templates

Click on **Charge Templates** in the menu to associate charges with frequently performed tasks, such as hybridizations, quality control and labeling.
A list of current templates will be displayed (see Figure 19).

Listing Charge Templates

Name	Description	Chips used	Chip cost	Labeling cost	Hybridization cost	Qc cost	Other cost	Edit	Destroy
Bioanalyzer	Bioanalyzer	1	5.0	0.0	0.0	0.0	0.0	Edit	Destroy
Hyb	Hyb	1	4.5	0.0	3.0	0.0	0.0	Edit	Destroy
One Cycle Labeling and Hyb	One Cycle Labeling and Hyb	1	4.5	1.25	2.0	0.0	0.0	Edit	Destroy
Two Cycle Labeling and Hyb	Two Cycle Labeling and Hyb	1	4.5	3.0	3.0	0.0	0.0	Edit	Destroy

New charge_template

Figure - 19 Current Charge Templates

To create a new template click on **New charge_template** and fill out the given fields then click **Create** (see Figure 20).

The **Name** identifies the template, the **Chips Used** refers to the number of chips to be charged for a particular template (if any), the **Default Description** is a short explanation of what the charges involve, and the **Chip cost** is the cost per chip listed under **Chips Used**. If a sample requires labeling the cost of that is listed under **Labeling cost**. If the sample is to be hybridized the cost of that will be listed under **Hybridization cost**. If the sample underwent QC, for example if the RNA, cRNA and/or fragmented cRNA were run on a Bioanalyzer, the cost would be listed under **Qc cost**. **Other cost** tallies all the other costs involved.

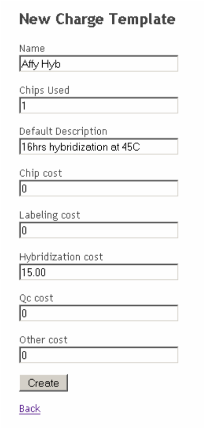

Figure - 20 Entering a New Charge Template

5.2 Charge Periods

Click on **Charges** and a list of all charge sets will be displayed, separated into charge periods (see Figure 21). A charge period includes all charges accumulated over a determined amount of time (usually a billing cycle).

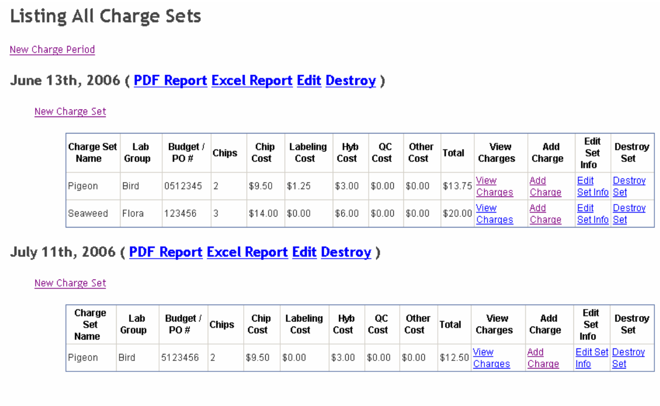
Listing All Charge Sets

New Charge Period

June 13th, 2006 (PDF Report Excel Report Edit Destroy)

New Charge Set

Charge Set Name	Lab Group	Budget / PO #	Chips	Chip Cost	Labeling Cost	Hyb Cost	QC Cost	Other Cost	Total	View Charges	Add Charge	Edit Set Info	Destroy Set
Pigeon	Bird	0512345	2	$9.50	$1.25	$3.00	$0.00	$0.00	$13.75	View Charges	Add Charge	Edit Set Info	Destroy Set
Seaweed	Flora	123456	3	$14.00	$0.00	$6.00	$0.00	$0.00	$20.00	View Charges	Add Charge	Edit Set Info	Destroy Set

July 11th, 2006 (PDF Report Excel Report Edit Destroy)

New Charge Set

Charge Set Name	Lab Group	Budget / PO #	Chips	Chip Cost	Labeling Cost	Hyb Cost	QC Cost	Other Cost	Total	View Charges	Add Charge	Edit Set Info	Destroy Set
Pigeon	Bird	5123456	2	$9.50	$0.00	$3.00	$0.00	$0.00	$12.50	View Charges	Add Charge	Edit Set Info	Destroy Set

Figure - 21 A Display of Charges Divided into Charge Periods, Subdivided into Charge Sets

A new charge period can be generated by clicking on **New Charge Period**. Name the charge period and click **Create** (see Figure 22).

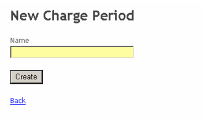

Figure - 22 Entering a New Charge Period

5.3 Charge Sets

Within each charge period are charge sets; charges divided into groups, each typically associated with a different lab group (see Figure 21). To list the charges

within a particular charge set click **View Charges**; a list of individual charges that a particular lab group has incurred will be displayed (see Figure 23).

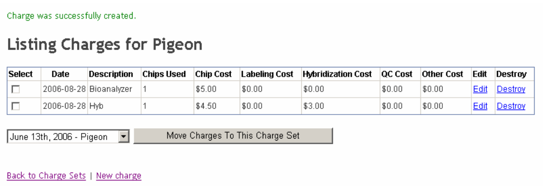

Figure - 23 Individual Charges in a Charge Set

There is an option to move individual charges into another charge set located in a different charge period. To move individual charges from one charge set to another, check the individual charges to be transferred and select the charge set to transfer them to from the drop down menu at the bottom of the table, then click **Move Charges To This Charge Set**. There is also an option to **Edit** and **Destroy** an individual charge.

To add a charge click on **New charge** and select an existing charge template from the drop down menu or fill out the fields, which are similar to those encountered when setting up a charge template. (see Figure 24).

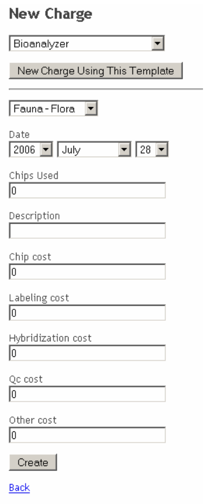

Figure - 24 Adding a New Charge

To get back to the listing of charge sets click on **Back to Charge Sets**. To add a new charge set click on **New Charge Set** (see Figure 25).

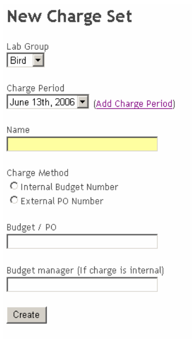

Figure - 25 Entering a New Charge Set

Select the lab group and charge period from the down menus. Enter a name for the charge set under **Name**, select the **Charge Method** and enter the Budget/PO number. When complete click **Create**.

6 Lab Groups

6.1 Creating and Changing lab groups

Click on **Lab Groups** to view a current list of lab groups (see Figure 26).

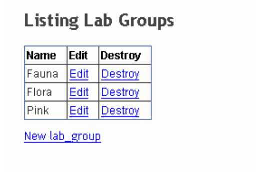

Figure - 26 Current Lab Groups

To change a lab group, click on **Edit** and make corrections. To delete a lab group click on **Destroy.** To add a new lab group, click on **New lab_group**; type the name of the lab group and click on the 'Create' button (see Figure 27).

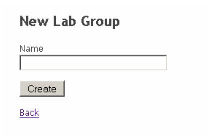

Figure - 27 Entering a New Lab Group

7 Exit SLIMarray

7.1 Log out

Click **Log Out** to exit SLIMarray and return to the login page.
